# Ultrasound Methods in the Evaluation of Atherosclerosis: From Pathophysiology to Clinic

**DOI:** 10.3390/biomedicines9040418

**Published:** 2021-04-13

**Authors:** Gabriel Cismaru, Teodora Serban, Alexandru Tirpe

**Affiliations:** 1 Fifth Department of Internal Medicine, Cardiology-Rehabilitation, Iuliu Hatieganu University of Medicine and Pharmacy, 400012 Cluj-Napoca, Romania; cismaru.gabriel@umfcluj.ro; 2 Medical Imaging Department, Iuliu Hatieganu University of Medicine and Pharmacy, 400162 Cluj-Napoca, Romania; serban.teodora8@gmail.com; 3 Research Center for Functional Genomics, Biomedicine and Translational Medicine, Iuliu Hatieganu University of Medicine and Pharmacy, 23 Marinescu Street, 400337 Cluj-Napoca, Romania

**Keywords:** atherosclerosis, ultrasound, CEUS, elastography, IVUS, VH-IVUS, atherosclerotic plaques, carotid, IMT

## Abstract

Atherosclerosis is a key pathological process that causes a plethora of pathologies, including coronary artery disease, peripheral artery disease, and ischemic stroke. The silent progression of the atherosclerotic disease prompts for new surveillance tools that can visualize, characterize, and provide a risk evaluation of the atherosclerotic plaque. Conventional ultrasound methods—bright (B)-mode US plus Doppler mode—provide a rapid, cost-efficient way to visualize an established plaque and give a rapid risk stratification of the patient through the Gray–Weale standardization—echolucent plaques with ≥50% stenosis have a significantly greater risk of ipsilateral stroke. Although rather disputed, the measurement of carotid intima-media thickness (C-IMT) may prove useful in identifying subclinical atherosclerosis. In addition, contrast-enhanced ultrasonography (CEUS) allows for a better image resolution and the visualization and quantification of plaque neovascularization, which has been correlated with future cardiovascular events. Newly emerging elastography techniques such as strain elastography and shear-wave elastography add a new dimension to this evaluation—the biomechanics of the arterial wall, which is altered in atherosclerosis. The invasive counterpart, intravascular ultrasound (IVUS), enables an individualized assessment of the anti-atherosclerotic therapies, as well as a direct risk assessment of these lesions through virtual histology IVUS.

## 1. Introduction

Atherosclerosis is a key multifactorial, systemic pathophysiological process that causes a plethora of pathologies such as coronary heart disease, peripheral arterial disease and ischemic stroke [1]. Although there are a number of effective therapies that target the underlying process and several prevention programs in place, atherosclerosis remains one of the central pillars that increase mortality worldwide [2]. One of the reasons for this wide distribution is the multitude and the heterogeneity of the risk factors that lead to atherosclerosis—hypertension, diabetes mellitus, and cigarette smoking among others [3], along with the typical silent progression of the atherosclerotic disease to advanced stages. Considering this silent progression, estimative risk stratification is essential in the current clinical practice through a direct assessment of the atherosclerotic plaque and determination of its vulnerability or indirectly, through statistically confirmed risk scores. Although far from perfect, clinical risk scores take into consideration several risk factors, including hypercholesterolemia, hypertension, cigarette smoking, and age—e.g., Systematic Coronary Risk Evaluation (SCORE) [4] and Prospective Cardiovascular Münster Study (PROCAM) [4].

One of the studies that highlighted the potential of ultrasound (US) methods in the direct evaluation of atherosclerosis is the 2015 prospective BioImage study [5]. It included 5808 asymptomatic adults (mean age: 69 years) and sought to assess vascular imaging biomarkers with predictive capabilities for early (3-year endpoint) atherothrombotic events. All patients were evaluated by coronary artery calcification (CAC) and 3D-carotid US with a quantitative 3D-US score calculated through summing the plaque areas from both carotid arteries (carotid plaque burden, CPB). Primary endpoint was considered to be major adverse cardiac events (MACE) that included an entity from the following spectrum: cardiovascular (CV) death—myocardial infarction—ischemic stroke. Secondary MACE endpoints included unstable angina, coronary revascularization, and all-cause death. On a 2.7-year median follow-up, 216 patients (4.2%) presented MACE, with 82 (1.5%) of the patients presenting primary events. MACE was found to increase with higher CPB and CAC. The authors concluded that imaging biomarkers that directly quantify atherosclerosis, including 3D-US, may be used as complementary methods to conventional risk factors, highlighting the importance of US techniques in the atherosclerotic plaque evaluation.

The current paper presents a critical, evidence-based review of the current US methods that can detect and characterize atherosclerosis, starting from the classic noninvasive bright (B)-mode US, contrast-enhanced ultrasound imaging (CEUS), elastography techniques, to the invasive spectrum—intravascular ultrasound (IVUS), transesophageal echocardiography, and epiaortic ultrasound. Furthermore, we include a brief chapter on pathophysiological considerations that are useful to the clinician who employs ultrasound methods in the evaluation of atherosclerosis. Our critical approach will keep in line the US differences between stable atherosclerotic plaques and vulnerable plaques, as the pathological risk for these entities is significantly different.

## 2. Brief Pathophysiological Considerations in Atherosclerosis: Why Is It Relevant?

The pathogenesis of the cardiovascular atherosclerotic disease is a chronic complex process, as atherosclerosis is a multifactorial disease. It involves hypercholesterolemia as the main trigger, along with a number of risk factors, including diabetes mellitus, hypertension, genetic abnormalities, and cigarette smoking, among others [3]. The present section discusses a brief perspective upon the pathophysiological implications of atherosclerosis that stands for the basis of a comprehensive view on the US evaluation of atherosclerosis.

**Figure 1:**
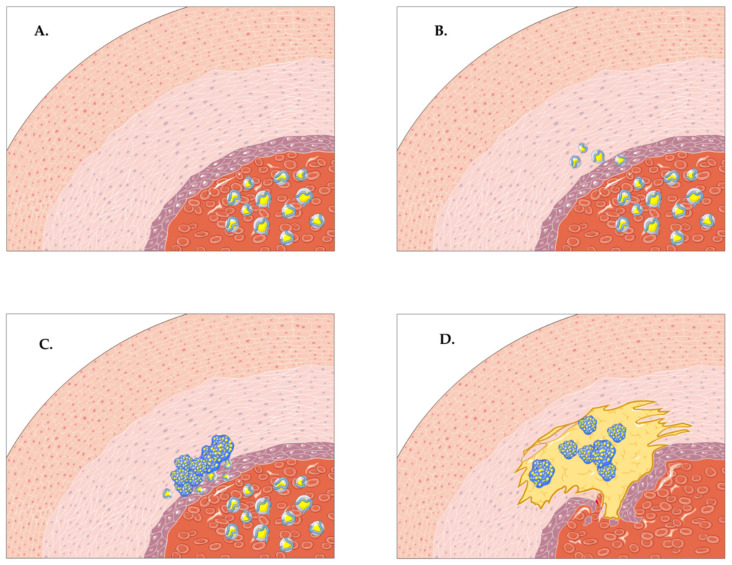
A brief overview of the pathogenesis of atherosclerosis. (**A**,**B**) The increased circulatory levels of low-density lipoprotein cholesterol (LDL-C) alter the permeability of the endothelium with a subsequent migration of the LDL-C according to the gradient—from the blood vessel to the arterial wall. (**C**) Tissue macrophages phagocytize and accumulate OX-LDL through scavenger receptors, forming foamy macrophages. (**D**) The increasing concentration of LDL-C and increasing uptake of LDL-C within the foamy macrophages gives birth to the atherosclerotic plaque with vulnerability features. The pro-atherogenic smooth muscle cells slowly migrate under the influence of local pro-inflammatory cytokines.

In healthy individuals, the equilibrium between the circulating cholesterol (Col) and the intracellular-endothelial Col ensures the homeostasis of low-density lipoprotein cholesterol (LDL-C). Atherosclerosis usually develops in the vasculature that displays low shear stress with altered blood flow—e.g., bifurcations and branch points [6,7]. In these specific areas, the endothelium switches to a pro-inflammatory state with a number of adaptive functions, including increased permeability and increased expression of adhesion molecules [8]. The increased level of plasma Col alters the endothelial permeability of the arterial wall, with a consecutive migration of LDL-C molecules within the arterial wall [3]. Vascular cell adhesion molecule-1 (VCAM-1) is selectively expressed by endothelial cells in response to the LDL-C migration, along P-selectin and E-selectin [9]. LDL-C is further oxidized to oxidized-LDL (OX-LDL)which has chemotactic properties [10]. Next, the monocyte recruitment through the arterial wall into the vascular intima takes place via diapedesis and is mediated by VCAM-1, P-selectin, and E-selectin [9,11]. There is also a concomitant migration of T lymphocytes within the vascular intima. OX-LDL is essential as it activates T lymphocytes through its antigenic character, while upregulating the adhesion molecules [11] and creating a vicious pathogenic circle. Next, the migrated monocytes differentiate to tissue macrophages. Macrophages are versatile immune cells with numerous functions [12] that, in this case, phagocytize and accumulate the OX-LDL through the scavenger receptors (specifically CD36, CD68, A and B1) [3], a notion that has been well known for decades [13]. The continuous uptake of OX-LDL in the subendothelial space leads to the formation of foamy macrophages (“foam cells”) and a progressive inhibition of macrophage migration [10]. When a critical point is reached due to the constant OX-LDL uptake, the foam cells undergo apoptosis and release pro-inflammatory mediators within the subendothelial area, increasing plaque vulnerability [14]. Concomitantly, smooth muscle cells (SMCs) are an essential player in atherogenesis. Their proliferation leads to the formation of the fibrous cap of the atherosclerotic lesion with inhibitory roles upon thrombosis and plaque rupture [15]. Although their supposed role is protective, the pro-inflammatory cytokines from the local environment lead to dramatic changes in the function of the SMCs, which develop a migratory macrophage-like phenotype and consequently become foam cells with pro-atherogenic abilities [16]. Figure 1 presents a brief overview upon the pathogenesis of atherosclerosis.

## 3. Noninvasive US Methods in the Evaluation of Atherosclerotic Plaques

Noninvasive US methods have gained increasing recognition for their ability and their large applicability. The present section provides an overview of the main noninvasive ultrasound techniques that can be employed in the characterization of atherosclerosis—B-mode US ± Doppler mode, contrast-enhanced ultrasound imaging, and elastography techniques. These methods mainly infer the examination of peripheral arteries if not otherwise specified.

### 3.1. B-Mode US and Doppler Mode

Conventional B-mode US has the ability to identify, locate, and characterize part of the spatial conformation of established atherosclerotic plaques. The addition of Doppler mode can provide an estimate of the flow and the velocities, allowing for a grading of the stenosis [17]. The conventional B-mode US plus Doppler mode is a powerful tool presenting a number of advantages, including its broad availability, cost-effectiveness, rapidity, absence of ionizing radiation and the possibility of re-examination [18]. However, the major limitations in applying B-mode US on a large scale for detecting atherosclerotic plaques are the depth of examination, obtaining adequate ultrasound windows for the superficial vessels that are taken into consideration, and the status of the plaque—there is limited use of US for incipient atherosclerosis. In general terms, vascular ultrasonography uses linear-array probes with a frequency around 5–8 MHz and has a limited capability for examining arterial vessels below a certain depth. The 2019 European Society of Cardiology (ESC) guidelines for the diagnosis and management of chronic coronary syndromes recommends (class IIa recommendation, level C) that carotid B-mode US can be performed in order to identify plaques in patients with suspicion of chronic coronary syndrome but with no known atherosclerotic disease [19].

**Figure 2:**
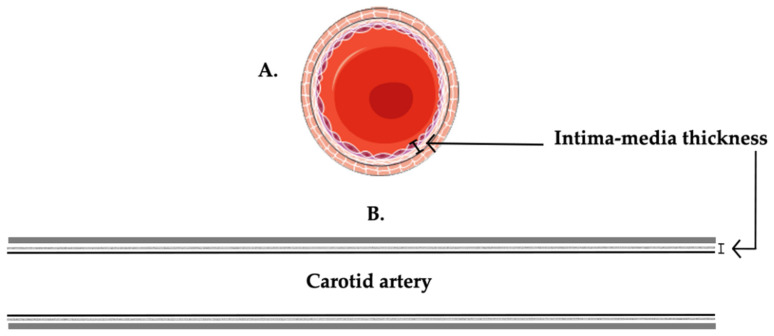
A schematic overview of the notion of IMT measurement. (**A**) Transversal section through the artery. (**B**) Longitudinal section through the artery. The main histological layers of the carotid artery are represented by the internal tunica intima, tunica media, and the outer layer adventitia.

In terms of utility, conventional B-mode US with Doppler mode is frequently used as a first-line examination in patients with recent cerebrovascular events in order to screen for atherosclerotic disease [20] in peripheral arteries. Furthermore, the 2019 ESC guidelines recommend (class IIb recommendation, level B) that the detection of atherosclerotic plaques by carotid US may be considered for asymptomatic patients as a risk modifier in CV risk assessment [19]. Carotid US has a similar risk prediction as coronary calcium score [5] and can be used in the screening of cardiovascular disease (CVD), taking into consideration the evidence-based recommendations. Carotid intima-media thickness (IMT) is an ultrasound-based quantitative parameter that may estimate a subclinical atherosclerotic disease in a given individual by summing the thickness of the two inner layers within the carotid artery—the intimal and medial layers (Figure 2). The normal values for adults range between 650 and 900 µm with an increase of 0–40 µm every year [21,22]. The IMT method has been developed by Pignoli et al. [23] and has been extensively used on large populations in order to evaluate CVD over the years, with a net increase in IMT in CVD patients along these studies [24,25,26,27,28,29]. Although a disputed entity, the IMT index has been studied in a great number of meta-analyses; furthermore, a negative association between high-density lipoprotein (HDL)-cholesterol and C-IMT has been established in the past [30]. A 1996 study by Cuspidi et al. [31] suggested that C-IMT index is more sensitive for vascular alterations due to hypertension rather than atherosclerotic plaques, rendering one of its leading limitations—precision. Although the measurement of the IMT index is inferior to direct atherosclerotic plaque detection and coronary artery calcium score in predicting CVD [32,33], and while its importance in the current clinical practice is rather debatable, it remains a pillar of the ultrasound atherosclerosis examination in patients with CVD. Currently, guidelines from the ESC do not recommend the use of carotid ultrasound IMT for screening and risk assessment in asymptomatic cardiovascular patients [19].

In advanced atherosclerosis, plaques may become visible on ultrasound examination, allowing for a direct morphological characterization. Although dependent on the examiner and their capabilities and prone to subjectivity, grading is also possible through standardized means using the Gray–Weale method [34]. A possible extension to include calcified and ulcerated plaques is feasible and more comprehensive—an example is the scale used in the Asymptomatic Carotid Stenosis and Risk of Stroke study [35]. In general terms, the Gray–Weale method classifies plaques into echogenic, predominantly echogenic, predominantly echolucent, and echolucent with potential additional classifications for calcified and ulcerated plaques. In a study by Huibers et al. [36], the authors sought to evaluate the potential predictive role of atherosclerotic plaque echolucency for ipsilateral ischemic stroke. The team found that patients with definitely echolucent plaques presented a significantly higher risk for ipsilateral stroke in the first 5 years after randomization [8.0%, 95% CI: 6.4%–9.6%], compared to patients with definitely nonecholucent plaques [3.1%, 95% CI: 2.1%–4.1%, *p* = 0.009]. Furthermore, after adjusting for other risk factors, Huibers et al. reported a 2.5-fold increase in ipsilateral stroke risk for patients with echolucent atherosclerotic plaques [hazard ratio, HR = 2.52, 95% CI: 1.20–5.25, *p* = 0.014]. Furthermore, a meta-analysis by Gupta et al. that included 7557 subjects over 7 analyzed studies found a statistically significant correlation between echolucent plaques (and not echogenic plaques) with all carotid stenosis severities (0–99%) taken into consideration, and the risk of future risk of ipsilateral stroke [relative risk, RR = 2.31, 95% CI: 1.58–3.39, *p* < 0.001]; when the stenosis was ≥ 50%, the RR for ipsilateral stroke increased [RR = 2.61, 95% CI: 1.47–4.63, *p* = 0.001], thus rendering an increased risk for echolucent plaques [37]. A study by Jashari et al. showed that vascular US presented a considerably high accuracy in identifying calcified atherosclerotic plaques with a volume over 8 mm^3^ with a sensitivity of 96%. However, the sensitivity of vascular US detection of calcified plaques decreased to 62% when the plaque volume was less than 8 mm^3^, with numerous false-negative results [38]. The Jashari study shows that US is a rather subjective method with several limitations in relation to the size of the plaque. Jashari et al. suggest that future US-based quantitative methods present potential and may improve the detection of atherosclerosis and lead to a better diagnostic capability, with the goal of achieving early plaque stabilization [38]. Moreover, US may also provide supplementary information regarding the plaque’s composition through the grayscale median (GSM) [39], a normalized parameter that can be repeated. GSM is a descriptor of plaque echogenicity; low GSM values are a characteristic of lipid plaques with a higher possibility of instability, while high GSM values are correlated with calcified atherosclerotic plaques and stable fibrotic plaques [40,41].

As previously mentioned, Doppler US can provide an estimate of the blood flow and the corresponding velocities, with a direct correlation to the grade of the stenosis, which are of significance for the planning of surgical/interventional therapies [17]. In addition, Doppler US can also investigate the arterial stiffness of the aorta through the measurement of the aortic pulse wave velocity (PWV), specifically from the common carotid artery to the common femoral artery or brachial–ankle PWV, which can be altered in the atherosclerotic disease [42]. Furthermore, a number of longitudinal studies show that the measurement of PWV is able to identify subclinical atherosclerosis, as well as to predict future cardiovascular events [43,44,45,46]. In a meta-analysis by Ohkuma et al. [47] that included 14673 participants without CVD, 1 standard deviation (SD, 3.85 m/s) increase in the brachial–ankle PWV was associated with a 1.19-fold increase in the risk of future CVD [HR = 1.19, 95% CI: 1.10–1.29, *p* < 0.001].

Although prone to subjectivity and highly dependent on the examiner, conventional B-mode US ± Doppler remains a powerful tool in assessing atherosclerosis. The constant development of technology will increase the accuracy of these methods, as further studies will need to standardize the information retrieved through these techniques.

### 3.2. Contrast-Enhanced Ultrasound Imaging: Adding a New Dimension

CEUS is a rather novel ultrasound-dependent technique that yields additional information compared to the classical B-mode US by using intravenous gas-filled microbubble ultrasound contrast agents (UCAs). These UCAs are blood pool agents that resonate with the ultrasound energy emitted by the examiner’s transducer and are subsequently destroyed by the ultrasonic waves, thus providing a better image quality and further quantifiable data [48]. There are a number of UCAs available on the market approved in both the European Union (EU) and the United States of America (USA) for the use of defining endocardial border/for left ventricular opacification—SonoVue/Lumason (Bracco Imaging SpA, Milan, Italy), Definity (Lantheus Medical Imaging, N Billerica, MA, USA), Optison (General Electric Healthcare, Princeton, NJ, USA).

CEUS can be used in numerous instances in CVD patients, from quantifying myocardial perfusion in stress echocardiography and detecting endoleaks after endovascular aortic aneurysm repair, to providing risk stratification in atherosclerosis and the surveillance of plaques during therapy. Specifically, in patients with atherosclerosis, CEUS allows for an in-depth characterization of the plaque “anatomy” through the assessment of plaque surface, ulceration, and neovasculature [49] and by providing a better resolution within the peripheral arteries.

**Figure 3:**
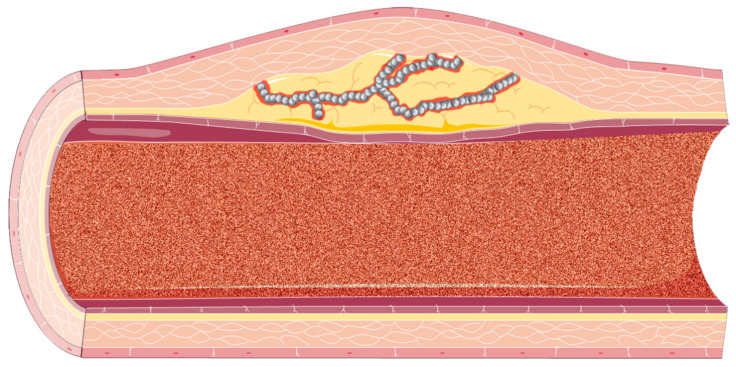
A schematic overview of the contrast-enhanced ultrasound imaging (CEUS) examination of the atherosclerotic neovasculature. The circulating ultrasound contrast agent (UCA) is represented within the arterial lumen as scattered noise, whilst the UCA microbubbles are represented as gross, gray bubbles within the atherosclerotic microvasculature.

As expected, the contrast agent administered during CEUS produces a luminal enhancement of the carotid artery displaying a hypoechoic tunica intima, hypoechoic tunica media, and hyperechoic tunica adventitia. This allows for a more in-depth evaluation of irregularities of the arterial wall, along with identification of hypodense plaques and ulcerations superimposed on atherosclerotic plaques [50]. Intuitively, this method allows for a better measurement of the C-IMT index by means of its enhanced resolution. In a study by Shah et al. that sought to compare the visualization of the IMT complex in 175 individuals with no known CVD, the images provided by CEUS were significantly superior to those obtained through conventional B-mode US. Specifically, in terms of percentage of IMT visualization, CEUS performed with 94% IMT visualization versus 61% for B-mode US for the right carotid artery (*p* < 0.001) and 95% versus 66% for the left carotid artery (*p* < 0.001), respectively [51]. Moreover, CEUS identified a larger number of atherosclerotic plaques compared to B-mode US (367 plaques versus 350 plaques, *p* = 0.02) [51]. Figure 3 presents a schematic overview of the use of CEUS in identifying plaque neovascularization.

Furthermore, as plaque neovasculature has been correlated with plaque instability [52] and increased microvessels within the atherosclerotic plaque has been shown to be a predictor of clinical outcome [53], CEUS presents a considerable advantage over conventional B-mode US. Several studies pointed that plaques with increased hypoechogenicity are more vulnerable to complications (i.e., increased risk for embolization with increased risk for ischemic stroke) and present increased neovascularization on CEUS examination [54,55]. The addition of UCAs allows for the development of quantification methods of the neovasculature—for example, a widely used visual scoring system with the following parameters: 0 (no visible UCA microbubbles within the plaque), 1 (moderate UCA microbubbles within the plaque), and 2 (extensive UCA microbubbles within the plaque) [53]. Other quantification methods include (but are not limited to) GSM, signal enhancement, arrival time, time to peak, semiautomatic quantification, and automatic quantification [53]. A study by Mantella et al. [56] on 459 stable patients sought to evaluate if vulnerable carotid atherosclerotic plaques are associated with significant coronary artery disease and future cardiovascular events through the CEUS evaluation of the plaque neovascularization. Mantella et al. found that the intraplaque neovascularization was able to predict significant CAD (≥50%) with a sensitivity of 92% and specificity of 89% using a cutoff score of 1.25 (area under the curve, AUC = 0.940), compared to maximum plaque height (MPH, AUC = 0.661), total plaque area (TPA, AUC = 0.665), and C-IMT (AUC = 0.625). Furthermore, in the same study, an intraplaque neovascularization score ≥1.25 was correlated with a higher incidence of future cardiovascular events (Kaplan–Meier analysis, *p* < 0.0001) [56], highlighting the predictive potential of CEUS.

As such, the new dimension brought by CEUS—the identification/gross quantitative assessment of plaque neovascularization—may prove efficient in patient risk stratification and prediction of future cardiovascular events, as well as in enhancing the power of conventional US where it is deemed necessary.

### 3.3. Atherosclerosis Assessment by Elastographic Techniques: Noninvasive Vascular Elastography (NIVE)

Elastography is a novel ultrasound technique considered by the European Federation for Ultrasound in Medicine and Biology (EFSUMB) guidelines to be a type of remote palpation, which enables direct assessment of different tissues. US elastography is based on the elastic properties of the material, consisting in the ability to restore their shape and size after being the subject of a deforming force. Elastography comprises semi-quantitative technologies, known as strain elastography (SE) and quantitative systems, represented by shear-wave elastography (SWE) [57].

In clinical practice, elastography techniques are increasingly useful in various circumstances and largely used for the evaluation of diffuse liver diseases [58]. Nonetheless, rising evidence suggests that US elastography allows adequate evaluation of the mechanical properties of plaques, displaying promising results for plaque vulnerability assessment [59,60]. Noninvasive vascular elastography (NIVE) is described as a semi-quantitative technology used for the appraisal of peripheral arterial wall biomechanics. It allows proper assessment of arterial wall movement induced by the natural cardiac pulse, recording time sequences of radiofrequency data [60]. Recent studies suggest that NIVE could be an advantageous substitute for magnetic resonance imaging (MRI), the current gold standard for plaque vulnerability analysis [61]. However, rising evidence suggests that the quantitative technique of acoustic radiation force impulse (ARFI) is suited for NIVE applications as well, improving the reproducibility of this procedure [62,63,64].

#### 3.3.1. Strain Imaging

Quasi-static elastography, or SE, makes use of either mechanically induced or active external displacement at different levels of the tissue surface. The result consists in the strain gradient, represented as an elasticity image entitled elastogram [57,65]. Considering that stable plaques are fibrocalcific, whereas vulnerable plaques are defined as thin fibrous cap atheromas, SE might represent a suited method for plaque characterization. In general terms, heterogeneous strain distribution suggests plaque instability [65].

A 2016 in vivo study showed that real-time elastography (RTE) provides good sensitivity (95.5%) and specificity (61.5%) of elastograms for identifying plaques with lipid-rich core. In human subjects, the Hansen study [66] detected (fibro)atheromatous plaques with 75% sensitivity and 86% specificity. Hansen et al. found no significant correlation between strain and parameters such as fibrous cap width, macrophage, or smooth muscle cell concentration. Subsequently, in a study that included 19 patients who underwent carotid endarterectomy, Liu et al. [67] found that RTE is able to describe the structure of carotid atherosclerotic plaques by discriminating between adipose tissue, fibrous tissue, calcifications, hemorrhage, and thrombosis. The Liu study provided a sensitivity of 50%, specificity of 100%, and accuracy of 89.4%, thus rendering RTE superior to conventional B-mode US. However, when both of these methods were combined, the overall sensitivity, specificity, and accuracy increased to 62.5%, 100%, and 94.7%, respectively [67]. Recent data reports provide similar results, with 71.6% sensitivity and 79.3% specificity for the combined assessment of carotid plaques [68]. In addition, Takimura et al. [69] proved that vascular elastography might be helpful for preoperative planning of endovascular treatment in occluded lower limb arteries.

However, some limitations of strain elastography must be taken into consideration. Even if the Cloutier study [68] found no significant difference between blood pressure and heart rate in each of the groups, these parameters are conventionally considered to be possible confounders for vascular elastography, along with the US frame rate. Additionally, plaque characteristics are not always unequivocal. In general terms, not all unstable plaques break and not all rupture-prone plaques present vulnerability features on histopathological assessment [70]. However, rising evidence suggests that US-based carotid elastography is a promising novel low-cost tool for plaque vulnerability assessment, with widespread availability, real-time capability, and great intra-observer reproducibility [71]. Even so, this noninvasive method requires further research on large populations in order to be implemented for screening, monitoring and follow-up [72,73].

#### 3.3.2. Shear-Wave Elastography

SWE is another category of elastography techniques. It makes use of a focused ultrasonic beam that generates shear waves by acoustic radiation force impulse [74]. The shear wave is traced through ultrafast plane acquisitions, whilst the velocity of the shear wave is determined and Young’s modulus calculated. As a general rule, the stiffer the tissue, the greater the amount of fibrosis and the higher Young’s modulus [57].

In fact, a recent systematic review by Pruijssen et al. [64] concluded that SWE is a feasible method to characterize atherosclerotic plaques with great reproducibility [75,76,77]. Displaying high sensitivity (87.1%), but low specificity (66.7%), SWE might play an important role in the multiparametric US assessment of plaque vulnerability [78] within peripheral arteries. The 19 included studies in the Pruijssen systematic review involved both human and non-human subjects, the latter consisting of animal arteries and polyvinyl phantoms. Pruijssen et al. found that SWE is able to evaluate plaque vulnerability with reference to symptoms, echogenicity on B-mode US and histology. However, SWE has several limitations, considering that quantitative SWE values differed significantly among subjects, especially in relation to patient and plaque characteristics. Of note is the Marais study [79] that measured shear-wave velocity in the anterior and the posterior wall of the carotid artery during the cardiac cycle. Marais et al. found that age and high blood pressure are independently associated with shear-wave speed in the anterior wall, but anterior wall SWE values did not differed significantly between normotensive and hypertensive subjects when compared at similar blood pressures. In contrast, posterior wall SWE values associated independently solely with age. Using the diameter values measured by echotracking, they were able to determine the new location of the carotid wall throughout the cardiac cycle, improving shear-wave acquisitions [79].

Several other studies reported that arterial stiffness correlates independently with age, genetics, hypertension, smoking, diabetes, and cardiovascular and renal diseases [80,81,82,83]. In the light of this, future studies should reduce the high heterogeneity among subjects and studies in order to establish proper cutoff values for the determination of unstable plaques [64].

Table 1 presents an overview of the advantages and limitations of the noninvasive ultrasound methods that have been approached in the current paper—conventional B-mode US + Doppler mode, CEUS, and vascular elastography.

## 4. Invasive US Methods: Where Are We?

Intravascular ultrasound is an invasive US technique that can directly visualize a given atherosclerotic lesion by using a miniaturized transducer incorporated within a 2.6 to 3.2 French/6 French catheter [110], assessing the extension of the lesion in both the longitudinal plane and in the axial plane [110]. Considering the fact that it was developed as a response to the limitations imposed by coronary angiography, IVUS is a more advanced method of imaging when compared to conventional angiography [111]. It provides several parameters such as the location of the plaque and its composition, the artery lumen size, and the absolute size of the arterial wall/vessel remodeling [112]. Furthermore, IVUS methods can be used by the interventional cardiologist for an accurate insight on complications during percutaneous coronary intervention (PCI), e.g., stent edge dissection or more tardive complications such as stent thrombosis [111]. IVUS currently uses compact, micro-scale transducers that are less than 1 mm in diameter. Although there is a tendency to increase the transducer frequency with a net result on increasing image resolution, current probes use variable frequencies, mostly between 20 and 40 MHz (center frequencies) and axial/lateral resolutions 60–200 μm/110–400 μm, respectively [113]. We highlight the importance of virtual histology (VH) IVUS, which is able to provide a classification of plaques through mathematically constructed tissue maps—fibrous, fibrofatty, necrotic core, calcific [114]. Grayscale and VH-IVUS techniques will be comprehensively reviewed within this chapter through a coronary atherosclerosis-dependent perspective.

### 4.1. Conventional Grayscale IVUS

The principle of B-mode IVUS is based on the electrical stimulation of the piezoelectric crystal found within the intravascular transducer. The piezoelectric crystal generates sound waves that propagate through tissues and are reflected in accordance with the acoustic properties of the material [115,116]. By this means, it enables real-time cross-sectional grayscale visualization of both morphological and pathological structures of the arterial wall [112]. Regarding atherosclerotic plaques, American College of Cardiology (ACC) provided a grayscale IVUS-based classification founded on their visual aspect (Table 2). However, due to IVUS’ inability to discern and quantify precise histologic components, these features are not suitable for accurate plaque evaluation [117]. Apart from that, conventional B-mode IVUS lacks reproducibility and accuracy. This comes as a result of the low resolution of this technique, together with the use of operator-dependent parameters, such as brightness and gain [118].

### 4.2. IVUS Shear Strain Elastography—Radiofrequency IVUS

**Figure 4:**
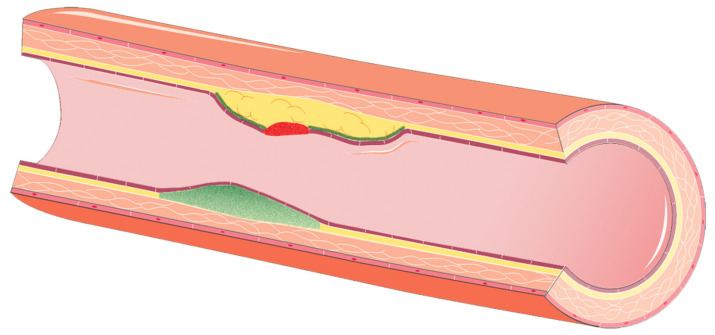
A schematic overview of the VH-IVUS potential findings on plaque examination within a given artery. The red tissue type is represented by calcified necrosis, while the dark green tissue consists of fibrous tissue. Fibrofatty plaques are represented by light green.

The implementation of IVUS-based post-processing methods utilizing radiofrequency data analysis and elastography overcame the constraints of qualitative visual interpretation of conventional grayscale IVUS [115,117,118]. Virtual histology IVUS (VH-IVUS) utilizes US backscattered signal to provide an accurate portrayal of plaque morphology into four major types, as shown in Table 3 [114,118,120]. The gross anatomy and VH-IVUS schematic representation of several atherosclerotic plaques are represented in Figure 4.

In clinical practice, VH-IVUS enables early detection and quantification of coronary atherosclerosis, serving as an accurate and reproducible technique for plaque vulnerability assessment. In addition, it seems to be an advantageous screening and monitoring tool that could help identify patients with increased risk [117]. In 2011, the virtual histology intravascular ultrasound in vulnerable atherosclerosis (VIVA) study was the first to report the correlation between thin cap fibroatheromas (TCFA) identified on VH-IVUS (VH-TCFA) and MACE. The VIVA study prospectively enrolled 170 patients with troponin-positive acute coronary syndrome (ACS) or stable angina, referred for percutaneous coronary intervention (PCI) and underwent 3-vessel VH-IVUS of the carotid arteries. Their results were confirmed by the Providing Regional Observations to Study Predictors of Events in the Coronary Tree (PROSPECT) study [121], performed on 697 patients with ACS who benefited from successful PCI for culprit lesions and were assessed by multimodality imaging of the carotid arteries. After a follow-up of 3 years, the PROSPECT study revealed that most of the non-culprit lesions associated with MACE were VH-TCFA or were characterized by a plaque burden greater than 70% and a minimal luminal area ≤ 4.0 mm^2^, highlighting the predictive capabilities of IVUS methods. Similar results were reported by the ATHEROREMO-IVUS study, conducted by Cheng et al. [122] on 581 subjects who underwent coronary angiography for stable angina or ACS. Their study established that VH-TCFA lesions in non-culprit coronary arteries are powerful predictive factors for the occurrence of MACE within the following year. In addition, they observed that larger VH-TCFA lesions carry an increased risk, in contrast to minor ones, particularly in the short term. All studies defined MACE as cardiovascular death, ACS, or unplanned coronary revascularization.

Another important aspect involving IVUS consists of its ability to detect minor changes within atherosclerotic plaques with substantial statistical power. Therefore, by using IVUS, smaller patient cohorts and considerable shorter trial periods are needed, significantly reducing expenses and facilitating the development of novel drugs [123]. For instance, in the Reversal of Atherosclerosis with Aggressive Lipid Lowering (REVERSAL) study [124], IVUS was able to uncover the complete remission of atherosclerotic plaques after 18 months of treatment with high-dose atorvastatin [117]. Similar results were reported by the Study of coronary Atheroma by Intravascular Ultrasound: the effect of rosuvastatin versus atorvastatin (SATURN) study [125], performed on 1039 patients with stable CAD. Of these, 71 subjects underwent serial IVUS evaluation for plaque composition changes under high-intensity statin treatment. The SATURN study concluded that statin therapy significantly reduced plaque size (percent atheroma volume: –1.6 ± 3.6%, *p* < 0.001) and vulnerability, the latter being a result of the increase in calcium tissue volume (from 1.2 to 2.1 mm^3^, *p* = 0.002) and a decrease in the fibrofatty one (from 23.1 to 13.4 mm^3^, *p* < 0.001). Subsequently, the IBIS-4 (Integrated Biomarker Imaging Study-4) study [126] explored the impact of 10 mg rosuvastatin on plaque constitution among 82 ST-elevation myocardial infarction (STEMI) patients with 146 non-culprit lesions appraised by IVUS. The investigators noticed that intensive rosuvastatin therapy led to dense calcium tissue enhancement and a decrease in fibrous tissue, whereas the necrotic core remained unchanged. Additionally, Lee et al. [127] indicate that aggressive lipid-lowering therapy significantly reduces the absolute plaque volume after only a period of 3 months, when compared with the control group. Recently, Kovarnik et al. [128] discovered that in spite of similar LDL-cholesterol levels, subjects with diabetes mellitus presented further atherosclerosis progression and additional locations with TCFA based on VH-IVUS examinations during aggressive lipid lowering treatment. Novel research focused on IVUS’ ability to evaluate short- and long-term consequences of drug-eluting stents, demonstrating that serial IVUS imaging can be a useful tool for monitoring therapy response [129,130].

#### Limitations and complications involving IVUS

Although IVUS produces a remarkably thorough examination of the vessel wall, it has several drawbacks that limit its everyday use. First and foremost, it is an invasive technique, always carrying a potential risk for vessel damage that requires an experienced operator [131]. Even if the implementation of radiofrequency IVUS exceeded the classical limitations of conventional grayscale IVUS, this technology is still far from being perfect. As previously mentioned, recent data doubts the capability of VH-IVUS to detect the necrotic core of atheromatous plaques [126]. In fact, a 2010 study by Thim et al. [132] found no association between the necrotic core volume atheromatous plaques, detected on VH-IVUS and the actual histology in porcine coronary arteries. Additionally, the GALOGOV trial [133] observed that that the supplementation of evolocumab in 968 subjects with stable CAD and ongoing treatment with maximal tolerable dose statin significantly reduced the atheromatous plaque volume, but did not improve the plaque composition when evaluated with VH-IVUS. This may be mainly due to a degree of VH-IVUS horizontal bias, which is unable to examine the precise segment during follow-up, since the patient’s heart rate varies at different time points [134]. Furthermore, Bourantas et al. [135] consider that plaque progression cannot be assessed solely by VH-IVUS and suggest that multimodality imaging should be used instead. Nevertheless, IVUS methods remain a powerful and precise method in analyzing atherosclerotic plaques.

### 4.3. The Use of Transesophageal Echocardiography for Aortic Atherosclerosis

Aortic atherosclerosis, carotid atherosclerosis, and atrial fibrillation are the main causes of cerebral and peripheral embolic events. In addition, aortic plaques are a marker of generalized atherosclerosis, its presence being an indirect sign of atherosclerosis in other territories, including lower limbs, renal, carotid, or coronary arteries. A number of studies [136,137] have shown that a plaque thickness of more than 4 mm can be used as a cutoff value to predict the occurrence of cerebral embolic events. Furthermore, aortic plaques ≥ 4 mm were detected as the most probable cause of stroke in one-third of patients with cryptogenic stroke. Other plaque-dependent risk factors that can increase the risk for cryptogenic stroke include the presence of ulcerations or mobile components associated with the plaque [138,139]. Among the most widely used methods of examining aortic plaques are computed tomography (CT), MRI, and transesophageal ultrasound. By far, transesophageal ultrasound is the most widely used due to its low cost, availability in almost any cardiology department, being free from ionizing radiation, and contrast agent administration [140].

**Figure 5:**
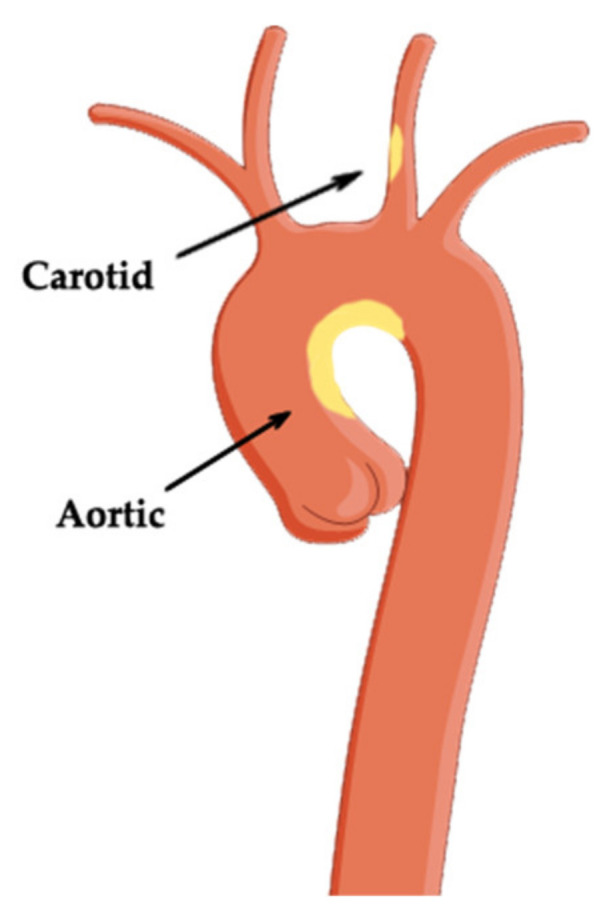
Atherosclerotic plaque of the aorta and carotid artery.

The practical importance of detecting atherosclerotic aortic plaques by transesophageal ultrasound is mainly validated in four clinical scenarios: (1) in patients with cryptogenic stroke, as an extensive assessment to detect the cause of cerebral embolism; (2) in patients who will be subjects of cardiac surgery and when manipulation of the ascending aorta is expected [141]; (3) in patients undergoing invasive cardiac procedures [142] when the aorta is the route to the left ventricle for guidewires or catheters: left accessory pathway ablation or left ventricle (LV) tachycardia, coronary angiography, ventriculography, or percutaneous implantation of the aortic valve [143], and (4) in patients with atrial fibrillation in whom the detection of atherosclerotic plaques brings an additional point to the CHA_2_DS_2_-VASc score [144,145], confirming the indication of long-term anticoagulation. Figure 5 presents a schematic view of aortic and carotid atherosclerotic plaques.

### 4.4. Epiaortic Imaging for Detecting Atherosclerosis

Epiaortic ultrasound was first used within a surgery in the 1970s to directly visualize the aortic valve and its function [146]. Over the years, its application extended. The presence of aortic atheromatosis is a marker of general atherosclerosis and prompts for an in-depth search by the surgeons in other areas such as carotids, coronary arteries, and peripheral arteries [147,148,149]. Atherosclerosis of the aorta is an independent factor of stroke comparable to thrombus in the left atrial appendage, carotid atherosclerosis, and atrial fibrillation [150]. The mechanism that can induce embolism in the presence of aortic plaques is direct during cardiac surgery—the manipulation of the aorta, introduction of the cannula, cross-clamping, and side clamping.

A study performed in 1996 found a higher risk of stroke with an odds ratio (OR) of 4.52 in patients with atheroma of the proximal aorta [150]. Van der Linden described precise locations of atherosclerosis that are related to an increased risk for stroke—atherosclerosis of the distal ascending aorta and the small curve of the aortic arch has an increased risk for stroke, whilst the proximal ascending aorta and the anterior surface of the aorta have no risk for stroke [151]. To detect the presence of aortic atheromatosis, surgeons rely on the palpation of the ascending aorta and thus determine the best site for cannulation, for cross-clamping, and for suturing [152]. However, there are several technical methods for assessing aortic atheromatosis, including transesophageal ultrasound and epiaortic ultrasound [146]. The traditional “gold standard” for intraoperative aortic atheroma detection is epiaortic ultrasound [153]. During the surgery, after the sternotomy, the transducer is placed directly on the aorta, which allows for a superior acoustic window and a superior performance to identify atherosclerotic plaques [154], with a smaller risk for embolization compared to manual palpation. A 5.7/7.5 MHz probe is inserted in a sterile sleeve with a small quantity of saline solution and then placed on the anterior surface of the aorta; the ascending aorta is scanned in transverse and longitudinal planes.

By far, epiaortic ultrasound is much more sensitive than manual palpation performed by some surgeons [154]. Furthermore, when compared to transesophageal ultrasound, epiaortic ultrasound seems to be more sensitive for identifying plaques [154]. It is important to note that the distal ascending aorta and the small curve of the aortic arch are difficult to assess by manual palpation, which may cause embolization, or by transesophageal echocardiography [146] due to the tracheal and right bronchus shadow.

#### Vulnerable Aortic Plaques

**Figure 6:**
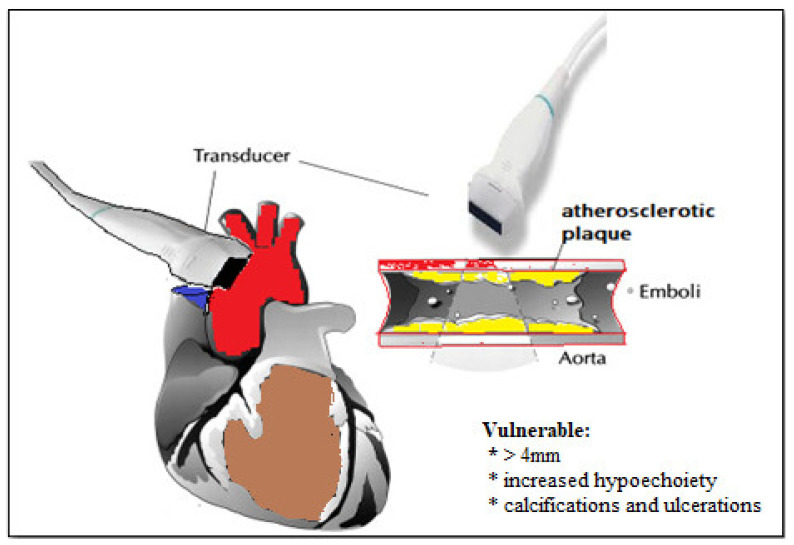
Vulnerable plaque seen in epiaortic ultrasound.

Similar to atherosclerotic plaques from coronary arteries, plaques from aorta have specific characteristics such as a precise location, thickness, morphology, and mobility that are associated with an increased risk [154]. Vulnerable plaques are considered those with thickness greater than 4 mm, with increased hypoechogenicity and mobility, as well as those that associate calcifications and ulcerations [154]. Figure 6 presents a schematic overview of vulnerable atherosclerotic plaques in epiaortic ultrasound.

Currently, there are guidelines that recommend the use of epiaortic ultrasound during surgical procedures performed on the ascending aorta [154]. If during the ultrasound an atheroma with dimensions of more than 3 mm thickness is detected, then the surgeon will use a specific approach that involves avoiding manipulating the aorta by hand.

## 5. Concluding Remarks

As atherosclerosis is a systemic pathology with silent progression, the early identification, characterization, and risk assessment is essential in the prevention chain. In the present review, we provided an integrative overview of both noninvasive (B-mode US + Doppler, CEUS, elastography) and invasive (IVUS, epiaortic) ultrasonography methods in the evaluation, characterization, and risk assessment of the atherosclerotic plaques. We have also highlighted the complexity of this pathological process by presenting a brief overview of the physiopathology of atherosclerosis, allowing for a better and comprehensive understanding of this pathology.

As expressed by several important studies such as the BioImage study, ultrasonography methods are a powerful addition to the clinician in the examination of atherosclerosis. In general terms, US techniques are rapid, cost-efficient, noninvasive, free from ionizing radiation and repeatable. Although the conventional B-mode US examination of atherosclerotic plaques lacks a proper standardization and the C-IMT index is a rather disputed parameter, this method can still be used to visualize, qualitatively assess plaques, and provide a rapid risk assessment. Furthermore, the addition of Doppler mode allows for a quantitative assessment of the stenosis through blood flow and velocity measurement, as well as an approximate assessment of subclinical atherosclerosis by arterial stiffness through the pulse wave velocity parameter. In addition, contrast-enhanced ultrasonography provides new features compared to B-mode US, allowing for an in-depth characterization and quantification of the plaque—from the anatomy to ulcerations and neovascularization. The importance of CEUS is highlighted by its ability to outline the neovascularization, which is a predictor of CAD and future cardiovascular events. Concomitantly, newly emerging elastography techniques may provide new quantifiable parameters that present a role in risk assessment and plaque vulnerability—i.e., the focal stiffness of the arterial wall in real time. The invasive counterpart of these methods—intravascular ultrasound, IVUS—empowers the physicians with the ability to directly visualize the plaques within a given artery. VH-IVUS performs best in the characterization of plaque components, thus allowing for an individualized assessment of the anti-atherosclerotic therapies, as well as a direct risk assessment of these lesions.

As such, these noninvasive and invasive US techniques can improve patients survival through a better disease-specific surveillance, diagnostics, as well as a better characterization of the atherosclerotic plaques and a more accurate risk stratification of the individuals with subclinical of clinically manifest atherosclerosis.

## Figures and Tables

**Table 1:** Overview of the clinical indications, validation status, advantages, and limitations of the discussed noninvasive US methods in the identification and characterization of atherosclerotic plaques and the evaluation of other cardiovascular pathologies.

Noninvasive US Method	Clinical Indications (Validation Status)	Advantages	Limitations
Conventional vascular B-mode US + Doppler mode	C-IMT measurement (validated) [84] Direct plaque visualization Provides GSM values (histopathological validation) [85] Doppler provides PWV values that estimate atherosclerosis (validated) [86] Extracranial (direct) and intracranial (indirect) vessel evaluation by Cervical Duplex US (validated) [87,88] B-flow and B-mode US for carotid fibromuscular dysplasia (unvalidated with catheter angiography) [89] Large vessel vasculitis diagnosis (unvalidated by histology) [90,91]	Noninvasive, rapid, widely available Cost-efficient Free from ionizing radiation Provides the possibility for re-examination Multiple Doppler modes (color, spectral, power) that can visualize and characterize the increased velocity within a stenosis [92] Doppler US can provide supplementary information through the grayscale median [39]	Limited depth of examination with the vascular probe Adequate ultrasound windows must be obtained for a thorough characterization Limited use for incipient atherosclerosis Limited use of IMT [19] Prone to subjectivity and dependent on the examiner
Vascular contrast-enhanced ultrasonography (CEUS)	Visualization and quantification of plaque neovascularization (histopathological validation) [93] Better C-IMT index measurement (validated) [94] Dissecans aneurysm: discernment between the true and the false lumen (validated using computed tomography angiography (CTA) [95] Abdominal aortic aneurysm: detection of intraluminal thrombus (validated using CTA) [96] Endovascular aortic aneurysm repair: identification and classification of endoleaks [97] Myocardial contrast echocardiography: quantification of myocardial perfusion, wall movement, and viability (unvalidated due to increased intra and interobserver variability) [49,98] Intracardiac thrombus characterization (validated by delayed-enhancement cardiac magnetic resonance) [99,100] Appraisal of vascularization within the vessel wall in large vessel vasculitis (unvalidated with histology) [101,102] Intra-cerebral vascular imaging (validated) [101,103]	Does not use ionizing radiation Cost-efficient, repeatable Provides quantifiable data [48] Provides better image quality and the delineation of the carotid lumen [48] Provides a better resolution for identifying atherosclerotic plaques and their anatomy—surface, ulceration, and neovascularization [53] Can provide risk stratification Can detect slow flow [104] UCAs are not nephrotoxic [104]	Uses intravenous contrast agents Requires specialized training Time limited (the concentration of UCAs decreases over a period of time—minutes) [104] Atypical artifacts: pseudo-enhancement artifact that may lead to the misinterpretation of results—non-linear propagation of the US waves [105] Artifacts: shadows produced by heavily calcified atherosclerotic plaques significantly hamper the examination of entities present within the acoustic shadow
Elastography techniques	Ultrasound strain imaging (histopathological validation after endarterectomy) [66] (validation using MRI) [106] Identification of lipid-rich atherosclerotic plaques [66] (Fibro)atheromatous plaque detection [66] Myocardial strain imaging: surveillance of adverse effects in cancer therapies (validated) [107,108] Diastolic wall strain: predictor of CVD [HR = 1.89, 95% CI: 1.04–3.36, *p* = 0.04] [109]	Adds a new dimension to the examination-the strain of the arterial wall/plaque [59] May discriminate between the adipose tissue, fibrous tissue, calcifications, hemorrhage and thrombosis [67] High reproducibility according to several studies [64,67]	Confounders SWE values can differ significantly among subjects in relation to patient and plaque characteristics [79] No standardized cutoff values

**Table 2:** American College of Cardiology clinical expert consensus regarding the qualitative assessment of atheromatous plaques by conventional B-mode intravascular ultrasound (IVUS) [115,119]

Category of Plaques	Conventional B-Mode IVUS Characteristics
Soft	The lesion echogenicity is lower than echogenicity of the surrounding adventitia
Fibrous	The atherosclerotic plaque has average echogenicity between soft echogenic plaques and highly echogenic calcified lesions
Calcified	The echogenicity is higher than the adventitia and is accompanied by acoustic shadowing
Mixed	Plaques contain more than one acoustic subtypes (>80% of plaques)

**Table 3:** Virtual histology IVUS (VH-IVUS): histology correlation of atherosclerotic plaques.

Tissue Type	Plaque Histology with Movat Pentachrome Stain	Color on VH-IVUS
Fibrous	Densely packed collagen	Dark green
Fibrofatty	Collagen with significant scattered lipid	Light green
Calcified necrosis	Foam cells, cholesterol clefts and microcalcifications	Red
Dense calcium	Calcium deposits lacking necrosis	White
